# Deep Learning for Neuromuscular Control of Vocal Source for Voice Production

**DOI:** 10.3390/app14020769

**Published:** 2024-01-16

**Authors:** Anil Palaparthi, Rishi K. Alluri, Ingo R. Titze

**Affiliations:** 1 Utah Center for Vocology, University of Utah, Salt Lake City, UT 84112, USA;; 2 School of Biological Sciences, University of Utah, Salt Lake City, UT 84112, USA;

**Keywords:** nonlinear control systems, artificial neural networks, voice production, speech acoustics, TensorFlow

## Abstract

A computational neuromuscular control system that generates lung pressure and three intrinsic laryngeal muscle activations (cricothyroid, thyroarytenoid, and lateral cricoarytenoid) to control the vocal source was developed. In the current study, *LeTalker*, a biophysical computational model of the vocal system was used as the physical plant. In the *LeTalker*, a three-mass vocal fold model was used to simulate self-sustained vocal fold oscillation. A constant/ǝ/vowel was used for the vocal tract shape. The trachea was modeled after MRI measurements. The neuromuscular control system generates control parameters to achieve four acoustic targets (fundamental frequency, sound pressure level, normalized spectral centroid, and signal-to-noise ratio) and four somatosensory targets (vocal fold length, and longitudinal fiber stress in the three vocal fold layers). The deep-learning-based control system comprises one acoustic feedforward controller and two feedback (acoustic and somatosensory) controllers. Fifty thousand steady speech signals were generated using the *LeTalker* for training the control system. The results demonstrated that the control system was able to generate the lung pressure and the three muscle activations such that the four acoustic and four somatosensory targets were reached with high accuracy. After training, the motor command corrections from the feedback controllers were minimal compared to the feedforward controller except for thyroarytenoid muscle activation.

## 1. Introduction

Voice production is a highly complex, fine motor skill that requires the coordinated function of three major components: lungs, larynx, and both sub-glottal (trachea) and supraglottal (vocal tract) airways. Lung pressure drives the airflow in the trachea towards the larynx. The activation of the intrinsic laryngeal muscles defines the posturing (adduction and tension) of the vocal folds present in the larynx. The airflow causes the vocal folds to oscillate under certain pre-phonatory vocal fold posturing conditions, generating audible pulses of airflow into the vocal tract. The vocal tract filters these pulses and radiates the sound into the atmosphere [1]. The control of voice production involves the complex integration of multiple types of information by the brain including acoustic, auditory, somatosensory, proprioceptive, and motor representations [2]. The frontal, temporal, and parietal lobes of the cerebral cortex, along with the subcortical structures such as the cerebellum, basal ganglia, brain stem and their functional connections form the voice motor control system [3]. Approximately 100 different muscles act on the three subdivisions of the vocal system (the lungs, larynx, and airway) [4]. In this study, we investigated the role of auditory and somatosensory–motor control of the intrinsic laryngeal muscles (vocal source) that control the pre-phonatory vocal fold posturing. The airway (both subglottal and supraglottal) shape was assumed constant during phonation. Lung pressure was considered a variable of interest for aerodynamical control, but the lungs’ muscular control was not included.

Elaborating on the neural control of the vocal system, in the human brain, the spatial and temporal auditory information is converted to perceptual information by the peripheral and central auditory nuclei [5,6]. More accurately, the physical properties of the acoustic pressures, i.e., fundamental frequency, sound pressure level, harmonic and noise content are transformed to corresponding auditory perceptual properties, i.e., pitch, loudness, brightness, and roughness. The planning for producing a sound with desired characteristics and the required laryngeal muscle activations are performed in cortical regions of the brain. These direct pathways can be called feedforward pathways. The auditory feedback of the produced phonation integrates with the planned information to generate auditory error maps. These maps are transformed to motor information in the motor cortex. Similarly, the proprioceptive and somatosensory information from the larynx is fed back to the motor areas via the somatosensory cortex and cerebellum [7,8]. The somatosensory error maps are also transformed into corrective motor commands in the motor cortex. The combined motor commands from the direct and feedback pathways in the motor cortex, gated by the basal ganglia, are projected on to the phonatory neurons that directly control laryngeal muscles. A detailed map of the brain regions involved in planning of auditory and somatosensory targets, feedback and descending pathways can be found in Jurgens (2002) [9].

In humans, the learning of vocal communication is dependent on the feedback of auditory and somatosensory information during development. As speech motor planning is honed, auditory feedforward control dominates production [2]. Auditory feedback, however, is required to maintain the quality and precision of speech throughout adulthood [5,6]. Evidence based on compensation to perturbation in auditory feedback and jaw movement indicate a key role for somatosensory feedback in both the learning and maintenance of speech [7]. The auditory and somatosensory feedback is essential to understand if the desired outcome was achieved through the feedforward control, and if not, to generate corrective motor commands such that the intended outcome is attained [10].

The majority of approaches to vocal source simulation to date have been open-loop. Physical parameters are specified, and acoustic output is produced. There is no internal recall schema for preferred paths through a maze of physical parameters. All explorations begin from scratch. Currently, there are a limited number of studies addressing the neural control of vocal source and vocal tract [11–14]. In particular, the DIVA neural control model for the vocal tract was recently expanded to include the control of the body-cover vocal source model (named LaDIVA) [15]. The acoustic targets for LaDIVA are the fundamental frequency (f_o_) and sound pressure level (SPL), and the control parameters are subglottal pressure (P_s_), and cricothyroid (CT) and thyroarytenoid (TA) muscle activations. Somatosensory targets are not included in the current version of LaDIVA.

The current study used a similar architecture to the DIVA model, i.e., one feedforward controller, one acoustic feedback controller, and one somatosensory feedback controller. However, the two differ in the composition of the controllers. The DIVA/LaDIVA model used linear Jacobian matrices to model the feedback controllers while the current study developed non-linear deep neural networks-based controllers, allowing more complex control mechanisms. Also, the current study included acoustic measures of voice quality (brightness and roughness) as acoustic targets, along with f_o_ and SPL. Control parameters included lateral cricoarytenoid muscle activation along with the lung pressure (P_L_), CT and TA activations. In contrast to the LaDIVA model, four somatosensory targets (vocal fold length, and fiber stress in the three vocal fold layers) were also included.

Machine learning and artificial intelligence are gaining traction in voice and speech research. They are being used for the automatic detection of voice disorders [16–18], improving the computational efficiency of simulators [19], and estimation of voice control parameters from acoustic output signals [20,21]. Currently, machine learning is being used to detect disorders such as laryngeal cancer, dysphonia, vocal fold nodules, polyps, edema, vocal fold paralysis, and neuromuscular disorders from voice and speech samples. Additionally, computationally expensive components of the traditional voice and speech production models such as the flow and pressure calculations are being replaced by faster machine learning models [19]. Unsupervised learning techniques are being used for disorder detection [22], emotion recognition [23], and voice quality detection using voice and speech samples [24].

Reinforcement learning is another branch of machine learning where the training data are not needed ahead of time [25]. The model learns to control the physical plant by interacting with it in a trial-and-error manner. The reinforcement learning approach can be used to learn the control of the vocal system. These neural control systems try to mimic how the brain controls the vocal system. When used with voice simulators, such neural control systems can provide insights into neuromuscular disorders such as vocal tremor, Parkinson’s disease, and spasmodic dysphonia. Even though the DIVA model, and other neural controllers of the vocal system, including the control system developed in the current study do not use reinforcement learning in its true sense, they fall under its broader category.

The primary objective of the current study was to develop a deep learning-based neural control system for the vocal source with feedforward and feedback controllers that generates lung pressure and intrinsic laryngeal muscle activation levels to achieve acoustic targets of interest. The secondary objective was to understand the relative role of the feedback and feedforward controllers towards the total motor command.

## 2. Materials and Methods

### 2.1. Control System

**Figure 1.**
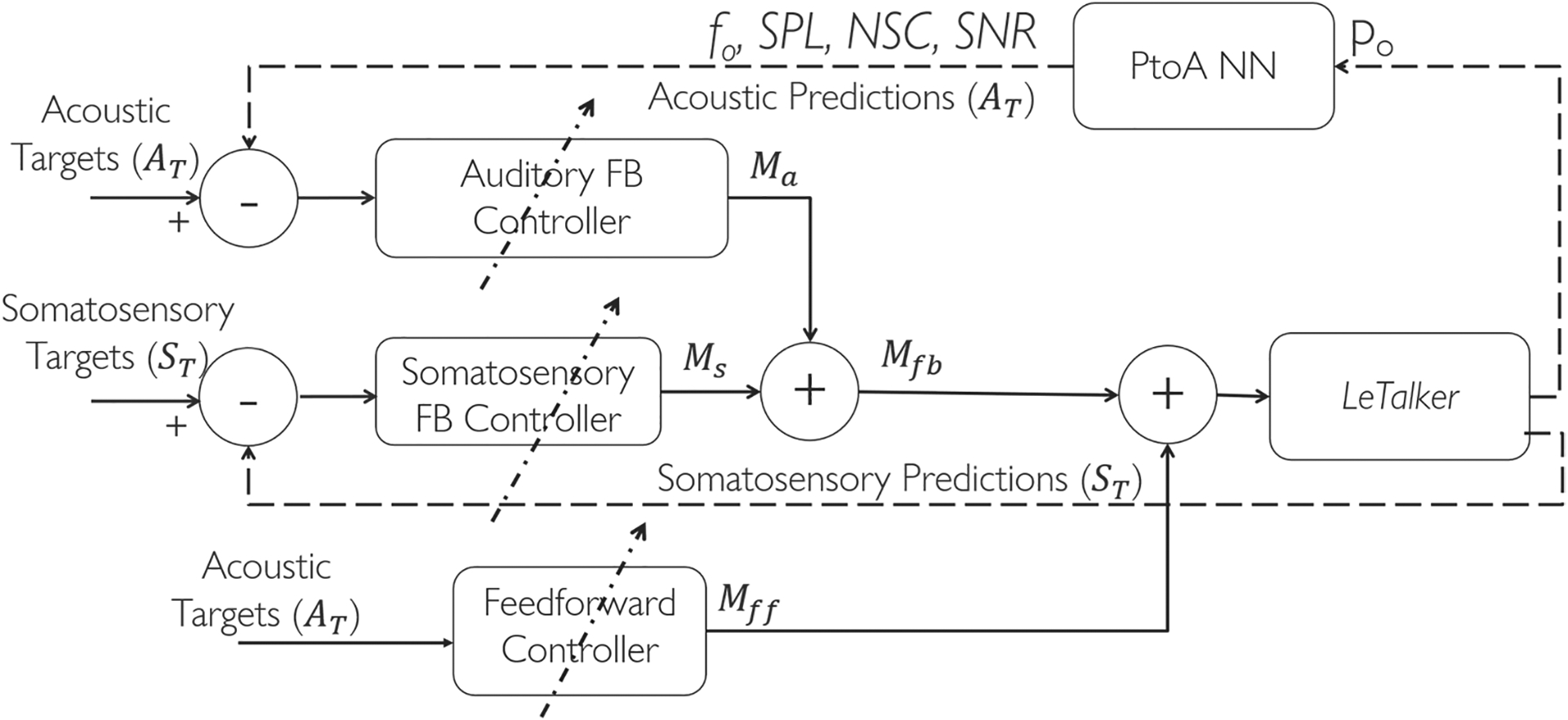
Neural Control System for the control of *LeTalker*.

The neural architecture designed in the current study to control the physical plant (*LeTalker*) is shown in Figure 1. Three controllers are used to control the *LeTalker*. The control begins with four auditory perceptual features, pitch, loudness, brightness, and roughness [26]. These perceptual features were expressed in terms of four acoustic physical quantities (fundamental frequency, sound pressure level, normalized spectral centroid, and signal-to-noise ratio, respectively) [27]. The pressure-to-acoustic converter neural network (PtoA NN), which was pretrained, estimates the corresponding acoustic features from the radiated pressure (P_o_) signal. The acoustic and somatosensory feedback controllers, respectively, receive the generated acoustic aspects of phonation and somatosensory states from the physical plant. The error between the targets and their corresponding predictions serves as input to the feedback controllers. The resulting motor commands from these negative feedback controllers minimize this error.

The feedback controllers, due to the nature of their inputs, suffer from delays in the feedback loop. Physiologically, this delay is compensated by auditory planning [28]. The feedforward controller represents the auditory planning in the higher-order brain regions and generates motor commands from auditory targets. In the current study, the feedforward controller was designed to take the acoustic targets as input and generate the feedforward motor commands. The output of the three controllers was added together to model the final phase of the sensory–motor transformation, i.e., produce motor commands. These commands were saturated between 0 and 1 before passing them as inputs to the physical plant.

### 2.2. LeTalker

A biophysical computational model (*LeTalker*) of the vocal system was used as the physical plant in the current study. The *LeTalker* takes lung pressure and three intrinsic laryngeal muscle activations (cricothyroid (CT), thyroarytenoid (TA), and lateral cricoarytenoid (LCA)) as inputs and generates the radiated pressure (P_o_) and the four somatosensory features as an output at every time sample. The four somatosensory features are the vocal fold length (L_g_), and the fiber stress (*σ*_*muc*_, *σ*_*lig*_, *σ*_*mus*_) in the three vocal fold layers (mucosa, vocal ligament, and TA muscle), respectively. Each phonation was 0.2 s long and simulated at a sampling rate of 44,100 Hz. A brief description of *LeTalker*’s dynamics is provided here; for a detailed explanation refer to [29–31].

#### 2.2.1. Three-Mass Vocal Fold Model

**Figure 2.**
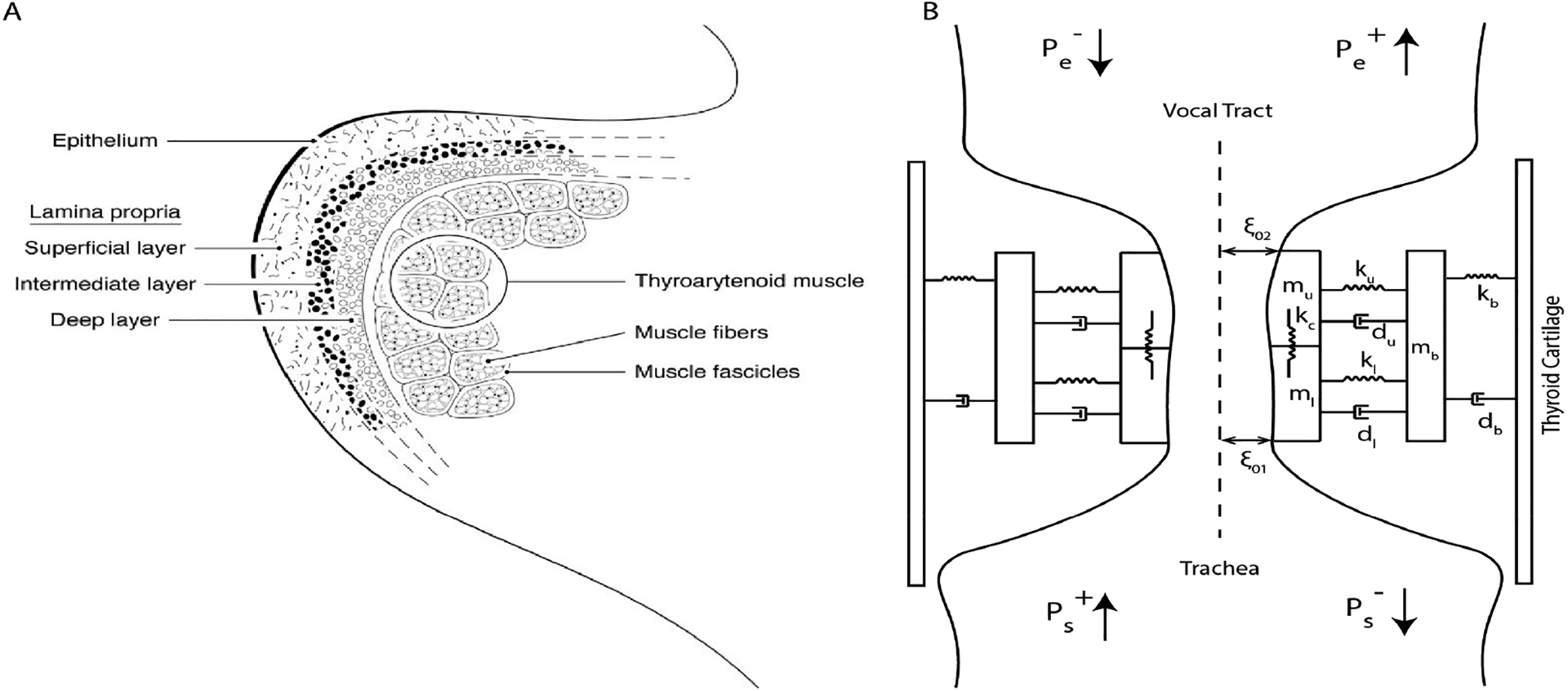
(**A**) The layered structure of the right vocal fold, (**B**) three-mass model approximation of the body-cover schema of the vocal folds.

Anatomically, each vocal fold is subdivided into five layers, as shown in Figure 2A. Computationally, the five layers are grouped either into two-layer schema (body (epithelium, superficial, and intermediate layers)-cover (deep layer and TA muscle)) or three-layer schema (mucosa (epithelium and superficial layers), vocal ligament (intermediate and deep layers), and TA muscle). In the *LeTalker*, the vocal fold dynamics were modeled using the two-layer schema and the three-layer schema was used to compute the fiber stress.

The three-mass model shown in Figure 2B is a lumped element approximation of the body-cover layer schema of the vocal folds. The two vocal folds were assumed to be symmetric and have the same movement. Male vocal fold dimensions of 1.6 cm length (L_0_), 0.8 cm thickness (T_0_), and 0.8 cm depth (D_0_) were considered. Two masses (*m*_*u*_ and *m*_*l*_) were used to model the cover layer, and the third mass (*m*_*b*_) simulated the effect of the body layer. The two masses of the cover layer were connected using a coupling spring. The coupling spring (*k*_*c*_) simulates the shear forces in the cover layer. Each of the cover layer masses was connected to the body layer mass using nonlinear springs (*k*_*u*_ and *k*_*l*_) and damping elements (*d*_*u*_ and *d*_*l*_). The two springs simulate the cover layer’s stiffness and the effective coupling stiffness between the cover and body layers. The body mass was connected to the rigid thyroid cartilage boundary using a spring (*k*_*b*_), which represent the effective stiffness of the body, and a damping element (*d*_*b*_). The equations of motion for the three masses were given in [31].

#### 2.2.2. Subglottal and Supraglottal Airway

The airway was modeled as a series of cylindrical sections. As the focus of the current study was on the neuromuscular control of the vocal source, the airway was kept constant without any articulation. The subglottal airway was modeled after MRI measurements [32] and consisted of 32 cylindrical sections. An/ǝ/vowel shape with 44 cylindrical sections was used for the supraglottal airway [33]. Each subglottal and supraglottal section was 0.3968 cm long [34], half the distance the sound travels in 1/44,100 s. The area function values for both subglottal and supraglottal airways were given in [35]. Attenuation factors were used to model the airway losses [29]. The choice of the section length allowed the computation of forward and backward traveling waves at section boundaries [34]. Radiation impedance was modeled by an inertance (L) and resistance (R) in parallel as in [36].

#### 2.2.3. Glottal Flow

The three-mass vocal fold model was connected to the subglottal and supraglottal airways. The glottal flow equation, *u*_*g*_ which is the primary vocal source can then be derived using the incident (P^+^) and reflected (P^−^) pressures at the glottis from the vocal tract. The computation of the glottal flow equation was described in [37]. Here, only the final equation was given:

(1) $$u_{g}=\left(\frac{a_{g}c}{k_{t}}\right)\left\{-\frac{a_{g}}{A^{*}}\pm {\left[{\left(\frac{a_{g}}{A^{*}}\right)}^{2}+\frac{4k_{t}}{\rho c^{2}}\left(P_{s}^{+}-P_{e}^{-}\right)\right]}^{1/2}\right\}$$

where *ρ* = 0.00114 g/cm^3^ is the air density, *c* is the speed of sound in air, *a*_*g*_ is the glottal area, $P_{s}^{+}$ is the forward traveling subglottal incident pressure on the glottis, and $P_{e}^{-}$ is the backward traveling supraglottal incident pressure on the glottis (see Figure 2B). If *A*_*s*_ is the subglottal area, and *A*_*e*_ is the supraglottal area, then the effective vocal tract area, A* was given as

(2) $$A^{*}=\frac{A_{s}A_{e}}{A_{s}+A_{e}}$$

and *k*_*t*_ as

(3) $$k_{t}=1-2\left(a_{g}/A_{e}\right)\left(1-a_{g}/A_{e}\right)$$

#### 2.2.4. Fiber Stress in the Vocal Fold Layers

The spring constants in the three-mass model can be augmented to account for anisotropic tension in the fibers present in the vocal fold layers. The body-cover representation of the vocal folds was divided into three layers (mucosa, vocal ligament, and thyroarytenoid muscle) to compute the fiber stress. The equations to compute the fiber stress in the three vocal fold layers were detailed in [31]. The passive stress in the three vocal fold layers was computed using the following equation

(4) $$\sigma_{p}=\{\begin{array}{cc} 0 & \text{for}\;\epsilon <\epsilon_{1},\\ -\frac{\sigma_{0}}{\epsilon_{1}}\left(\epsilon -\epsilon_{1}\right) & \text{for}\;\epsilon_{1}\leq \epsilon \leq \epsilon_{2},\\ -\frac{\sigma_{0}}{\epsilon_{1}}\left(\epsilon -\epsilon_{1}\right)+\sigma_{2}\left(e^{C\left(\epsilon -\epsilon_{2}\right)}-C\left(\epsilon -\epsilon_{2}\right)-1\right) & \text{for}\;\epsilon >\epsilon_{2}. \end{array}$$

The mucosa and ligament layers have only passive stress, whereas the TA muscle has both active and passive stress. Therefore, the fiber stress, *σ*_*mus*_ in the TA muscle layer was given as

(5) $$\sigma_{\text{mus}}=a_{TA}\sigma_{\text{am}}\text{max}\left(0,1-b{\left(\epsilon -\epsilon_{m}\right)}^{2}\right)+\sigma_{p}$$

where *ϵ* is the vocal fold strain, *ϵ*_1_ is the strain where the linear portion of the passive stress goes to zero, *ϵ*_2_ is the strain where the exponential part begins, *σ*_0_ is the stress when *ϵ* = 0, *σ*_2_ is a scale factor for the exponential portion, *a*_*TA*_ is the thyroarytenoid muscle activation level between 0 and 1, *σ*_*am*_ = 105 kPa is the maximum active stress in the TA muscle, *ϵ*_*m*_ = 0.4 is the optimum sarcomere strain, and *b* = 1.07 is the empirically determined constant for each muscle. Table 1 provides the parameter values used in the current study to compute the fiber stress in the three vocal fold layers. Except where suggested, the parameters in Table 1 do not have any units.

#### 2.2.5. Rules for Muscle Control of the Three-Mass Model

Vocal fold posturing is defined as the adduction, abduction, elongation, or shortening of the vocal folds resulting from the activation of intrinsic laryngeal muscles. In this section, the rules/equations that govern vocal fold posturing originally derived in [31] are presented. To reduce the number of control parameters, only three muscle activations were used: cricothyroid (CT), thyroarytenoid (TA), and lateral cricoarynteoid (LCA). The LCA muscle can approximate the role of the other two muscles (interarytenoid, and posterior cricoarytenoid).

##### Vocal Fold Elongation Rule

(6) $$\epsilon =G\left(R_{T}a_{CT}-a_{TA}\right)-Ha_{LC}$$

where *G* = 0.2 is the gain of elongation, *R*_*T*_ = 3.0 is the torque ratio, *H* = 0.2 is the adductory strain factor, and *a*_*CT*_, *a*_*TA*_, *a*_*LC*_ are the CT, TA, and LCA muscle activation levels, respectively, between 0 and 1.

##### Dynamic Vocal Fold Length, Thickness, and Depth Rule

(7) $$\text{Length},L_{g}=L_{0}(1+\epsilon )$$

(8) $$\text{Thickness},T=\frac{T_{0}}{1+0.8\epsilon }$$

(9) $$\text{Depth}\;\text{of}\;\text{body}\;\text{layer},D_{b}=\frac{a_{TA}D_{\text{mus}}+0.5D_{\text{lig}}}{1+0.2\epsilon }$$

(10) $$\text{Depth}\;\text{of}\;\text{cover}\;\text{layer},D_{c}=\frac{D_{\text{muc}}+0.5D_{\text{lig}}}{1+0.2\epsilon }$$

where *D*_*muc*_ = 0.2 cm, *D*_*lig*_ = 0.2 cm, and *D*_*mus*_ = 0.4 cm are the depth of the mucosa, vocal ligament, and TA muscle layers, respectively.

##### Vocal Fold Adduction Rule

The adduction rule for the glottal half-width *ξ*_02_ at the vocal processes was governed by the LCA muscle activation.

(11) $$\xi_{02}=0.25L_{0}\left(1-2a_{LC}\right)$$

##### Vocal Fold Convergence Rule

The TA muscle governs the prephonatory convergence of the glottis.

(12) $$\xi_{c}=T\left(0.05-0.15a_{TA}\right)$$

**Figure 3.**
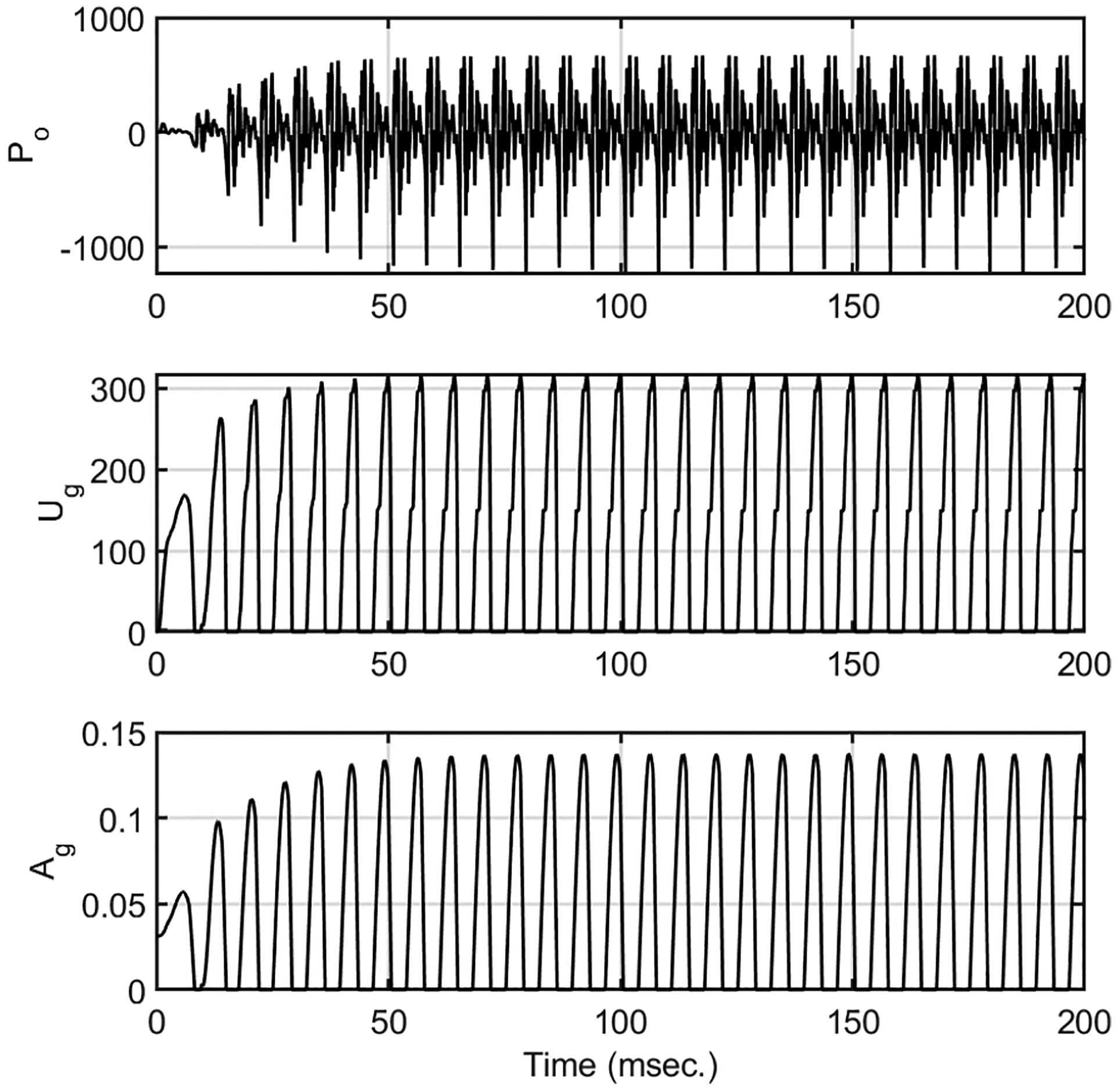
Example waveforms generated by *LeTalker*. (**Top**) radiated pressure, (**Middle**) glottal flow, and (**Bottom**) glottal area signals.

These rules, governed by the intrinsic laryngeal muscle activations, change the adduction and tension in the vocal folds. They, in turn, change the dynamics of vocal fold vibration, resulting in several acoustic characteristics in the sound. Figure 3 shows example radiated pressure (P_o_), glottal flow (u_g_), and glottal area signals (a_g_) generated by the *LeTalker*. The muscle activation plots of the acoustic and somatosensory features with respect to their dominant control parameters were given in [35].

### 2.3. Neural Network Architecture

**Figure 4.**
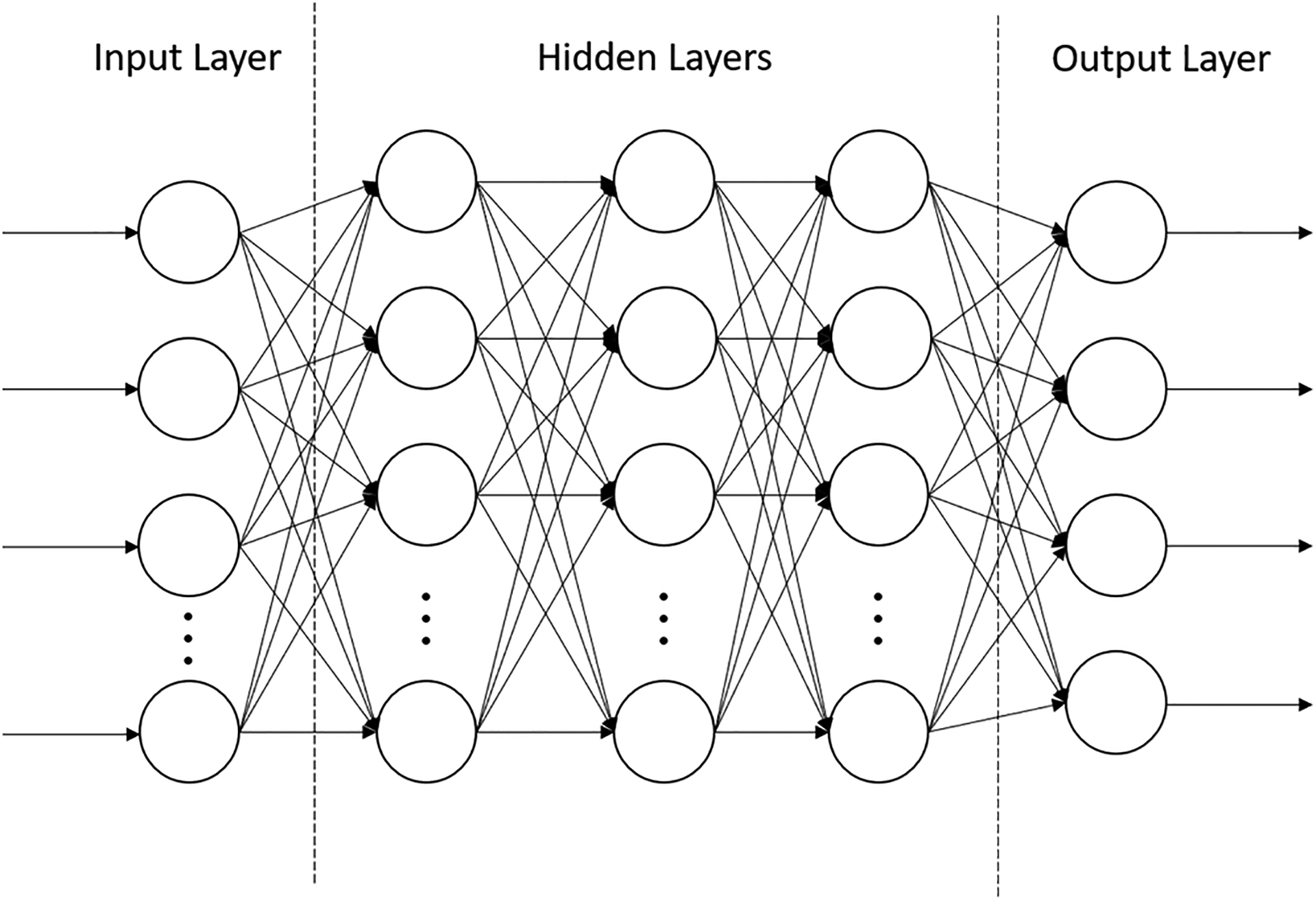
Structure of the neural networks used in the architecture designed for the control of *LeTalker*.

The feedforward and feedback controllers, along with the PtoA NN, consisted of three parts: 1. an input layer with the same number of cells as the number of inputs; 2. a core with the hidden layers; and 3. a full connected output layer with the same number of cells as the number of output parameters. Figure 4 shows the structure of the neural networks.

The auditory and somatosensory feedback controllers receive error information, i.e., the difference between targets and corresponding predictions and transform them into lung pressure and muscle activations. Therefore, the input layer consists of four inputs, the core consists of three layers of 128 neurons each, and the output layer consists of four cells corresponding to the lung pressure and the three muscle activations. Similarly, the feedforward controller uses a four-cell input layer corresponding to the four acoustic planned targets and the output layer has four cells corresponding to the lung pressure and the three muscle states. The core of the acoustic feedforward controller consists of three layers of 256 neurons each. The number of layers and units used in the layers was empirically determined. All the layers of the feedback controllers use a hyperbolic tangent activation function with a −1 to 1 range. This allows for the feedback controllers to compensate for the error in motor commands in both positive and negative directions. All the layers of the feedforward controller use a sigmoid activation function allowing the control outputs to vary between their range of 0 and 1.

The *LeTalker* generates one radiated pressure value at every time sample. A buffer was maintained to store 1102 radiated pressure values (i.e., 25 ms of sound output at 44,100 Hz sampling rate). The buffer was updated at every time sample using a 1-sample sliding window of 25 ms. The PtoA NN takes this buffer of 1102 radiated pressure values as input and generates four acoustic predictions as output at every time sample. Therefore, the PtoA NN has 1102 neurons in the input layer, 4 neurons in the output layer, and 512 neurons in each of the hidden layers.

### 2.4. Training and Testing the Neural Networks

To train and test the control system, 50,000 radiated pressure signals with a length of 0.2 s each were generated by varying the lung pressure and the three muscle activations with Monte Carlo random sampling. The *P*_*L*_ and *a*_*LC*_ were generated using normal distributions, whereas *a*_*CT*_ and *a*_*TA*_ were generated using uniform distributions. The set of these 50,000 control parameters (*M*_*T*_) were given as input to *LeTalker*, and the corresponding steady-state somatosensory and acoustic features were computed. Among them, a set of 40,000 features were used as targets for training, and the remaining 10,000 were used as targets during testing of the controllers. The entire set of 40,000 acoustic and somatosensory targets were used simultaneously to train the controllers. The acoustic features were transformed into a normal distribution and the fiber stress were transformed into a logarithmic scale. The weights for all the three controllers were updated simultaneously at every time sample. The targets were kept constant for the 0.2 s of simulation without any shuffling. The cost function to update the weights was the mean-squared error between the targets and their predictions as the targets were all continuous variables:

(13) $$\text{cost}=\frac{1}{N}\sum_{1}^{N}{\left[\left(M_{T},S_{T},A_{T}\right)-\left(M_{P},S_{P},A_{P}\right)\right]}^{2}$$

where *M*_*T*_, *S*_*T*_, *A*_*T*_ are the control, somatosensory, and acoustic targets, respectively, and *M*_*P*_, *S*_*P*_, *A*_*P*_ are the current control, somatosensory, and acoustic predictions, respectively. An Adam optimizer with a learning rate of 5 × 10^−4^ was used for updating the weights. Since all the N = 40,000 targets were used simultaneously, only one run of 0.2 s was used to train the controllers. As a result, the weights were updated 8820 times (0.2 s of simulation at 44,100 Hz sampling rate).

Each of the 50,000 radiated pressure signals was divided into 50 segments, each with a length of 25 ms (1102 samples) to train the PtoA NN. This was accomplished by using a 25 ms sliding window with a step size of 3.5 ms. Thus, the 50,000 signals were split into 2.5 million segments. For each of the 2.5 million segments, corresponding acoustic features were computed. Among the 2.5 million segments, 1.5 million were used to train, and the remaining 1 million were used to test the PtoA NN. An Adam optimizer with a learning rate of 1 × 10^−3^ was used to update the PtoA NN weights. The training was run for 800 epochs. The cost function to train the PtoA NN was the mean-squared error between the targets and predictions, as given below:

(14) $$\text{cost}=\frac{1}{N}\sum_{n=1}^{N}{\left(A_{T}-A_{P}\right)}^{2}$$

where *A*_*T*_ are the acoustic targets, *A*_*P*_ are the acoustic predictions, and *N* is the number of training segments. The trained models were tested on the data that were not used for training using mean-squared error, mean absolute error, and error in % metrics.

## 3. Results

### 3.1. PtoA Neural Network

**Figure 5.**
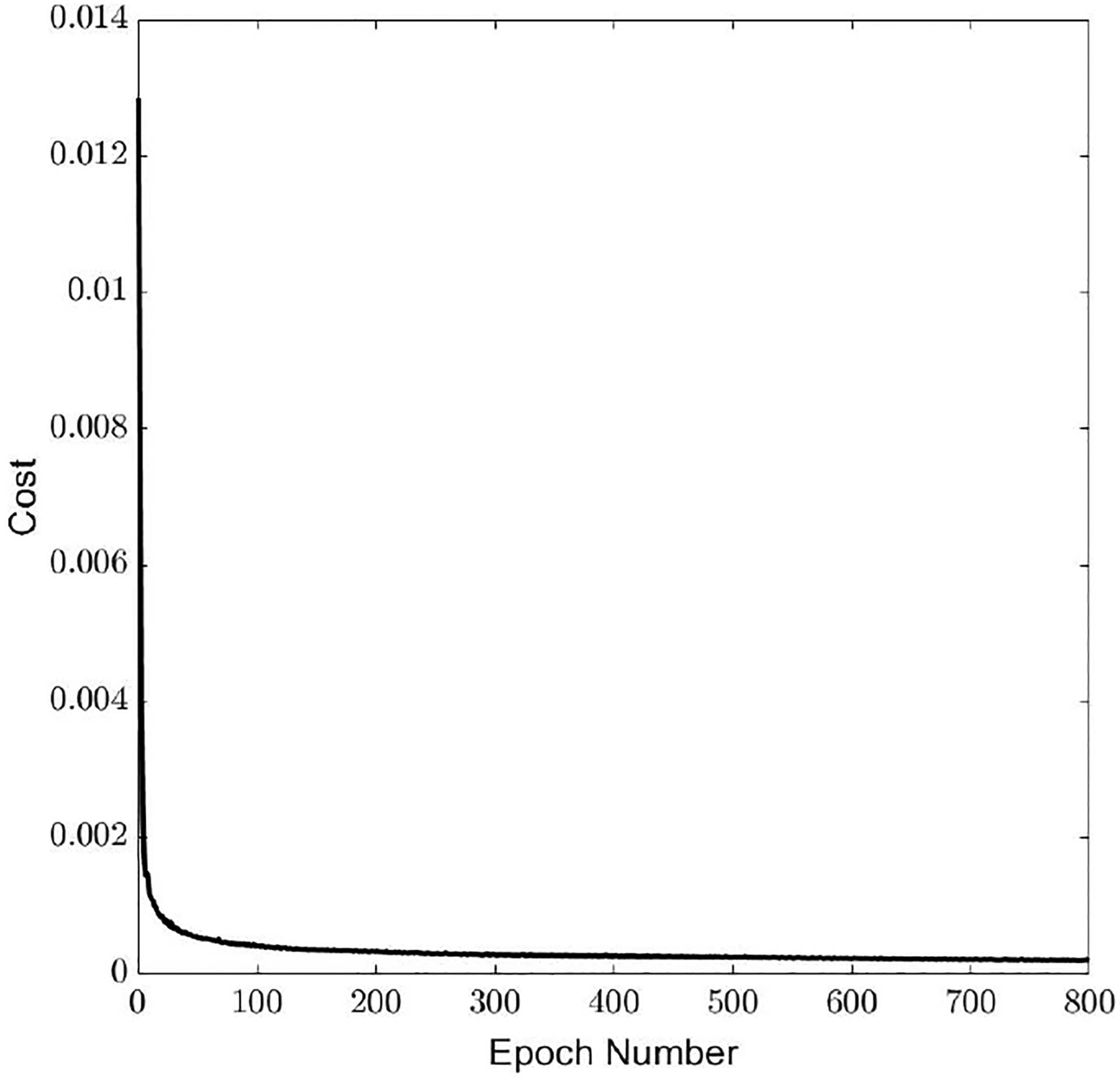
Cost function used to train PtoA NN as a function of epoch number.

**Figure 6.**
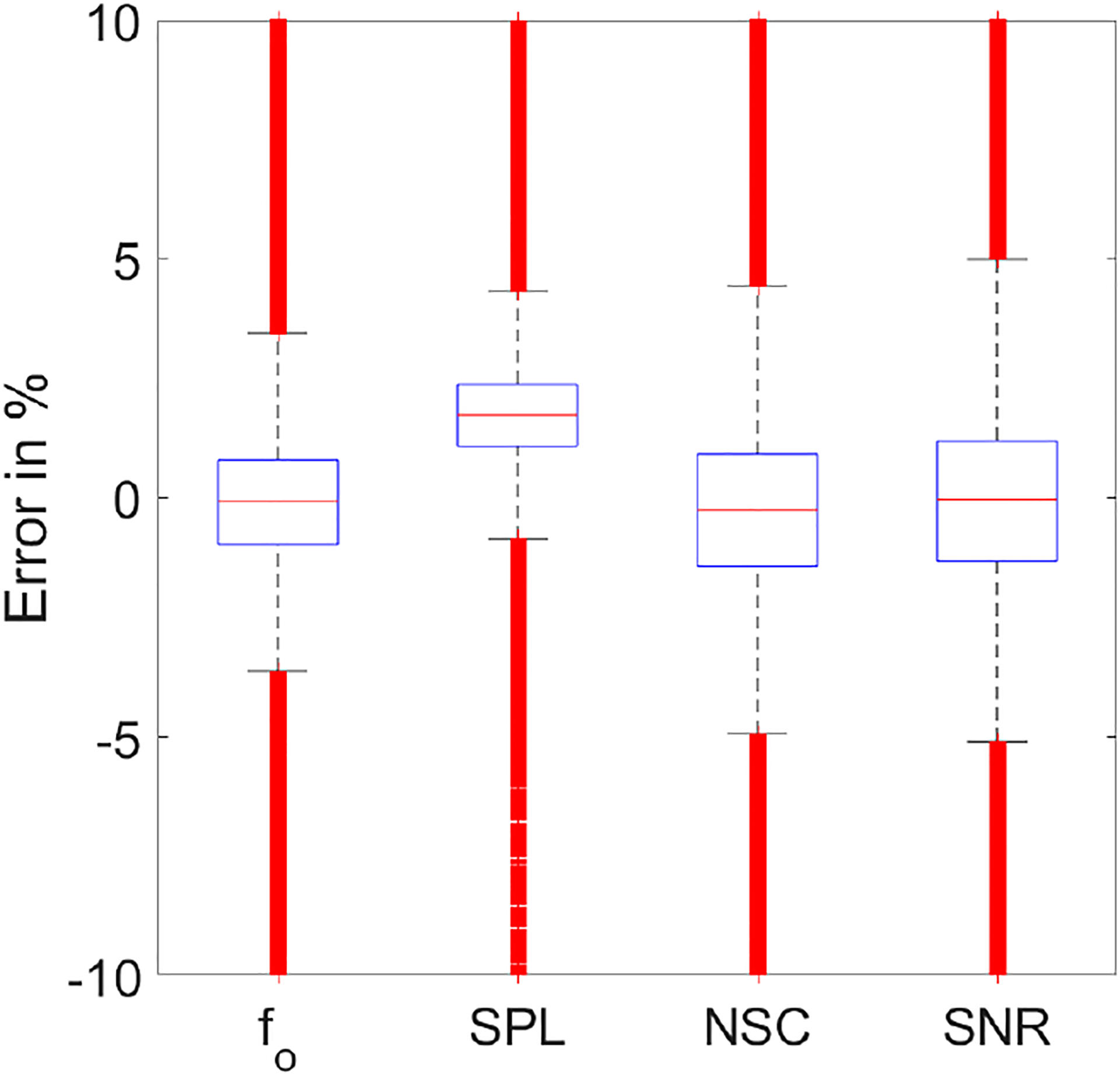
Boxplots showing error in % for the four acoustic features from the PtoA NN test data.

The change in cost function during training as a function of the number of epochs is shown in Figure 5. The training cost reduced to 2.07 × 10^−4^ in the normalized units by the end of the training with an accuracy of 0.96. On the test data, the cost was 4.03 × 10^−4^, with an accuracy of 0.94. Boxplots for the percentage error computed based on Equation (15) for all the four acoustic features from the test data are shown in Figure 6.

(15) $$\text{Error}\;\text{in}\%=\frac{A_{T}-A_{P}}{A_{T}}\times 100$$

For better clarity, the *y*-axis was limited to ±10%. However, the outliers extended up to −500% and +100%. A value was considered an outlier if it was away more than 1.5 times the interquartile range from the box’s edges. The percentage of outliers with respect to the total test segments for f_o_, SPL, NSC, and SNR were obtained as 6.9%, 2.7%, 3.8%, and 8.6%, respectively. The percentage error for all four acoustic features varied between ±6%, excluding the outliers (Figure 6). The median error was close to 0% for all the features except SPL, whose median error was at 1.7%. This indicates that the predicted value of SPL is often less than the target. The range of error was higher for NSC and SNR compared to that of f_o_ and SPL. This was expected given the abstractness of brightness and roughness the NSC and SNR represent, respectively.

**Figure 7.**
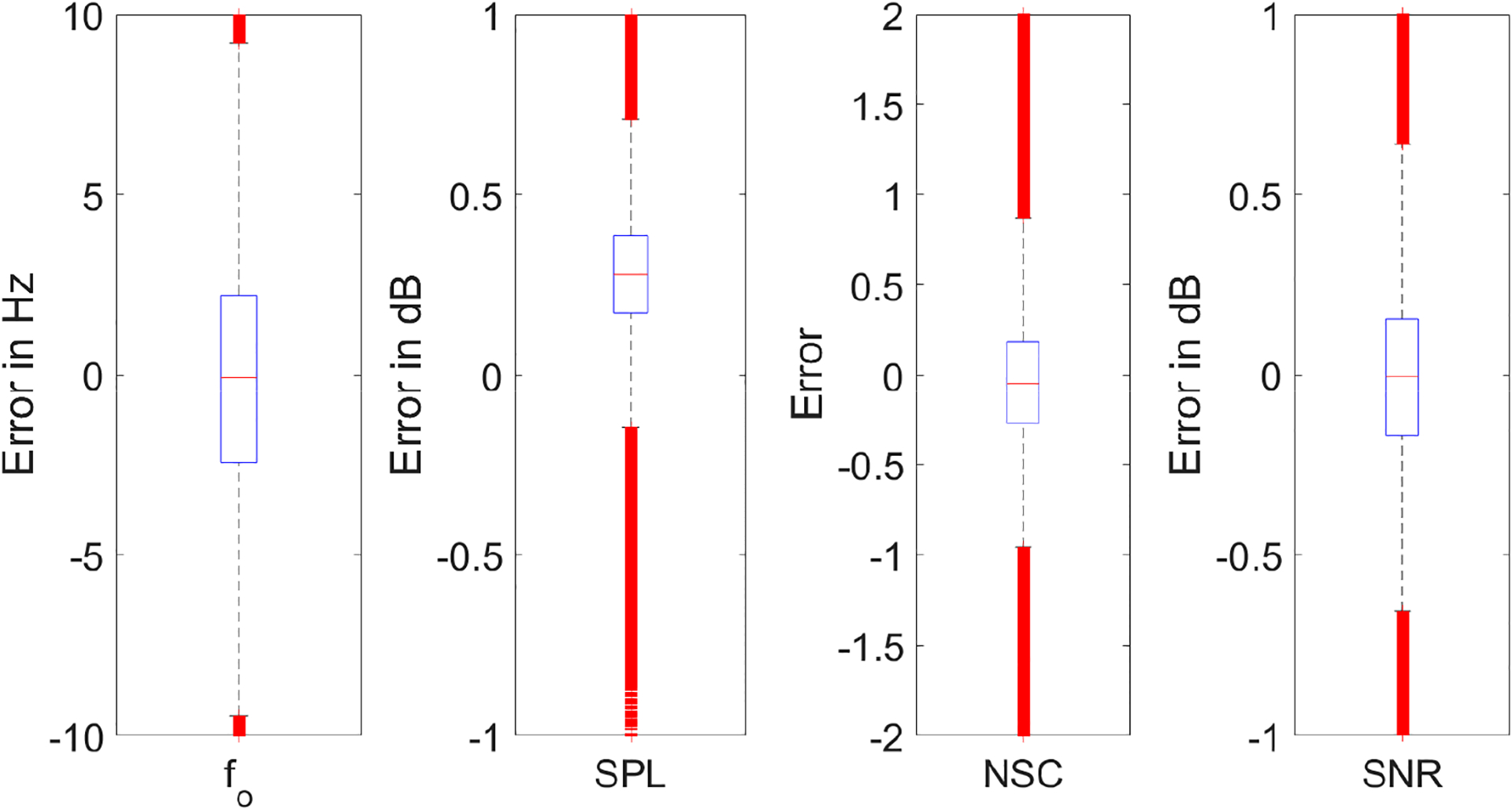
Difference between targets and predictions for the four acoustic features from PtoA NN test data in actual units.

Figure 7 shows the boxplots for the difference between targets and predictions in the actual units of the acoustic features from the 1 million test segments. It can be observed that the error was between ±10 Hz for f_o_, ±1 dB for SPL, ±1 for NSC, and ±1 dB for SNR, excluding the outliers. The ranges for f_o_, SPL, NSC, and SNR across all the 2.5 millions segments were 350 Hz, 60 dB, 66 (no units), and 124 dB, respectively. This suggests that the prediction error obtained was only a tiny fraction of the target values for most cases, indicating that the PtoA NN can accurately predict the acoustic features for a wide variety of sound signals.

### 3.2. Control System

**Figure 8.**
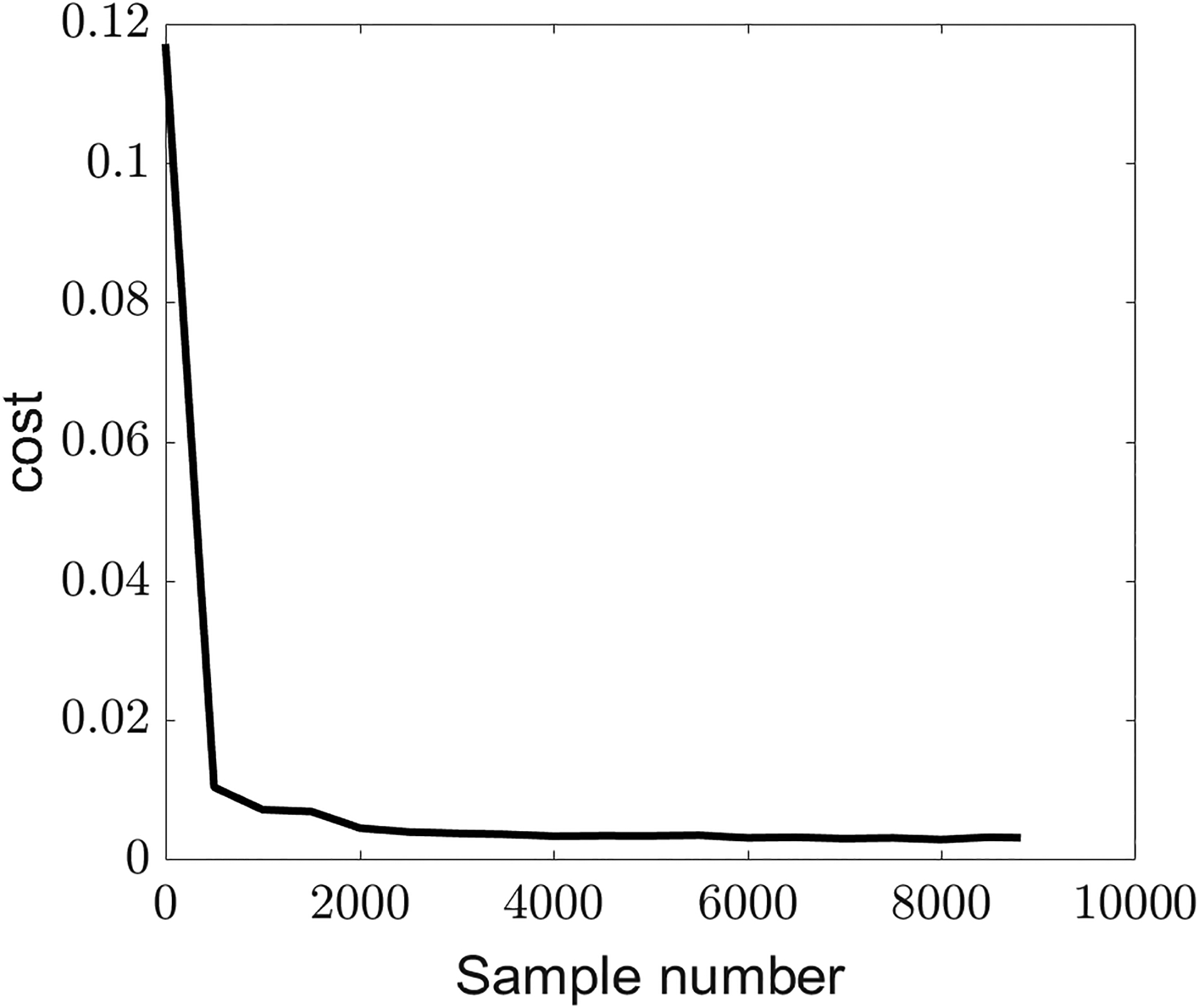
Cost value used to train the control system as a function of sample (epoch) number.

Figure 8 shows how the cost function given in Equation (13) was reduced as a function of the epoch (sample) number for the training data. The cost reduced to 0.0032 in normalized units at the end of the 8820 epochs. The error did not distribute equally across the three types of features/parameters. The individual cost values in normalized units for control, somatosensory, and acoustic targets were obtained as 0.0067, 0.0019, and 0.0011, respectively, at the end of the training. The error was highest for predicting muscle activations. Physiologically, a set of somatosensory and acoustic features could be achieved by many combinations of muscle activations, described as motor equivalence. Therefore, higher errors in muscle states does not necessarily imply error in reaching the targets but the presence of alternate activations that result in the same somatosensory and acoustic targets. A combined cost value of 0.0041 was obtained for the test data in normalized units at the end of 0.2 s of simulation. The individual cost values in normalized units for muscle, somatosensory, and acoustic features from the test data were obtained as 0.0076, 0.0021, and 0.0027, respectively, at the end of the 0.2 s of simulation. This indicates that the performance decreased on the test data, but only slightly. Here, as well, the major error occurred in reaching the muscle targets.

**Figure 9.**
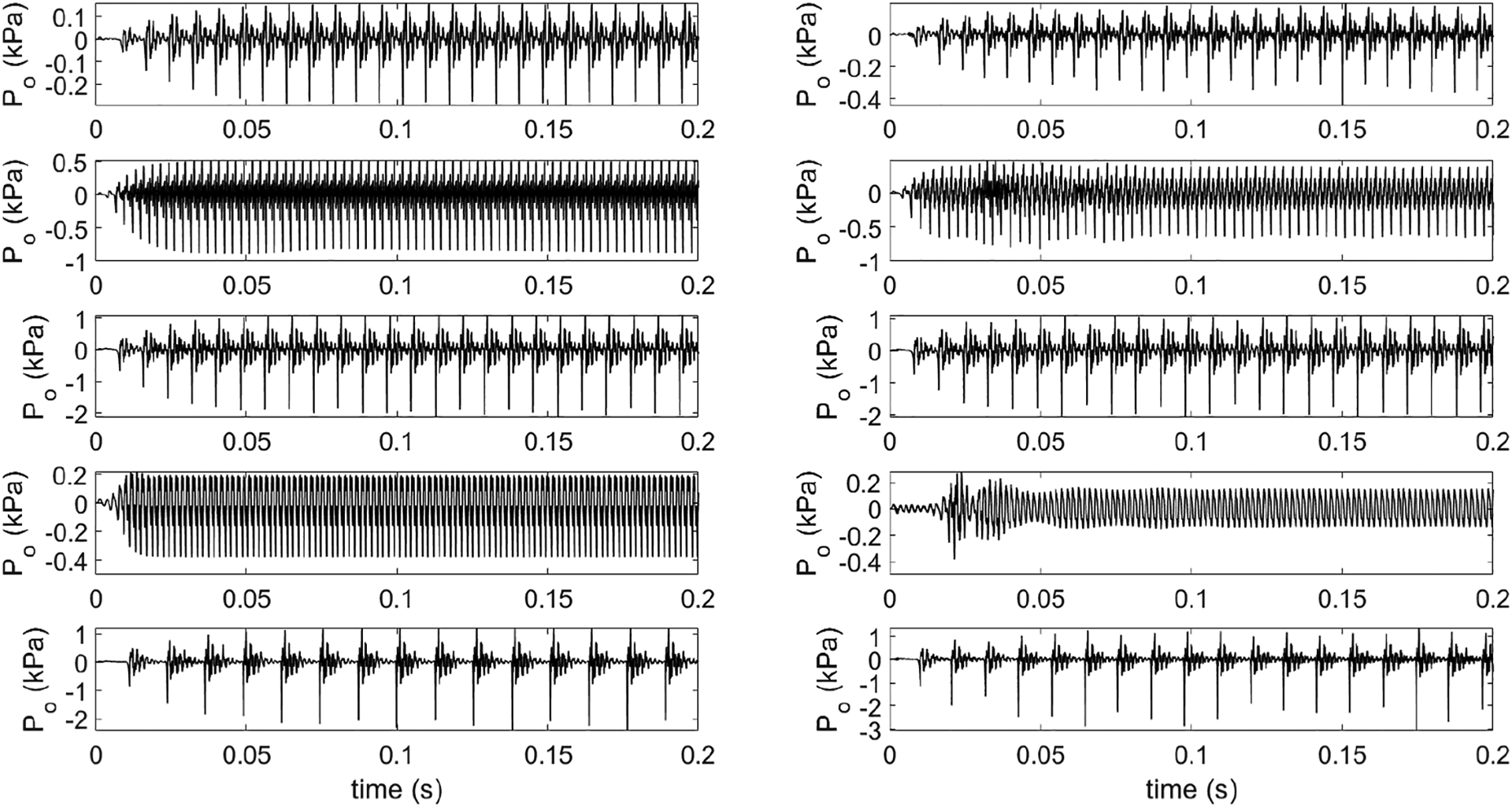
Comparison between targets (left) and *LeTalker* predictions (right) from the test set. (**First row**) A typical signal in the speech range. (**Second row**) A signal with high f_o_. (**Third row**) A signal with high SPL. (**Fourth row**) A signal with low spectral centroid. (**Fifth row**) A signal with higher noise.

Figure 9 shows the example target and predicted radiated pressure (sound) signals generated by *LeTalker* for some of the test data cases. The first row shows the pressure signal with an f_o_ in the speech range, the second row shows a signal with a higher f_o_, the third row shows the waveforms with higher SPL, the fourth row shows the waveforms with a lower spectral centroid, and the fifth row shows the waveforms with more noise in the signal. From the waveforms, it can be observed that the predicted signals reached the steady state between 40 and 80 ms, typically seen in *LeTalker*. The predicted radiated pressure signals obtained by the control of neural architecture were very similar to the targeted signals. However, there were slight differences between the targets and the predicted signals. The difference is apparent in the pitch and amplitude of the signals. For example, the f_o_ is lower in the predicted signal compared to the targeted signal (second row), and the amplitude is lower in the predicted signal compared to the targeted signal (fourth row). The differences in other two features cannot be observed easily from the waveforms. Therefore, the targeted features and their corresponding predictions for these five examples are given in Table 2.

From Table 2, it can be observed that for the muscle parameters, a significant difference occurred in reaching LCA activation (*a*_*LC*_). There were slight differences in the prediction of *a*_*CT*_ and *a*_*TA*_ as well (first and fifth row). Lung pressure was predicted accurately for all five examples. With regard to somatosensory features, the differences are small for all the four features. For the acoustic features, there are significant differences in some cases for all the features. These results indicate that across all the test cases, the error would be higher in the prediction of *a*_*LC*_, and moderately high in the prediction of *a*_*CT*_, *a*_*TA*_ and the four acoustic features.

**Figure 10.**
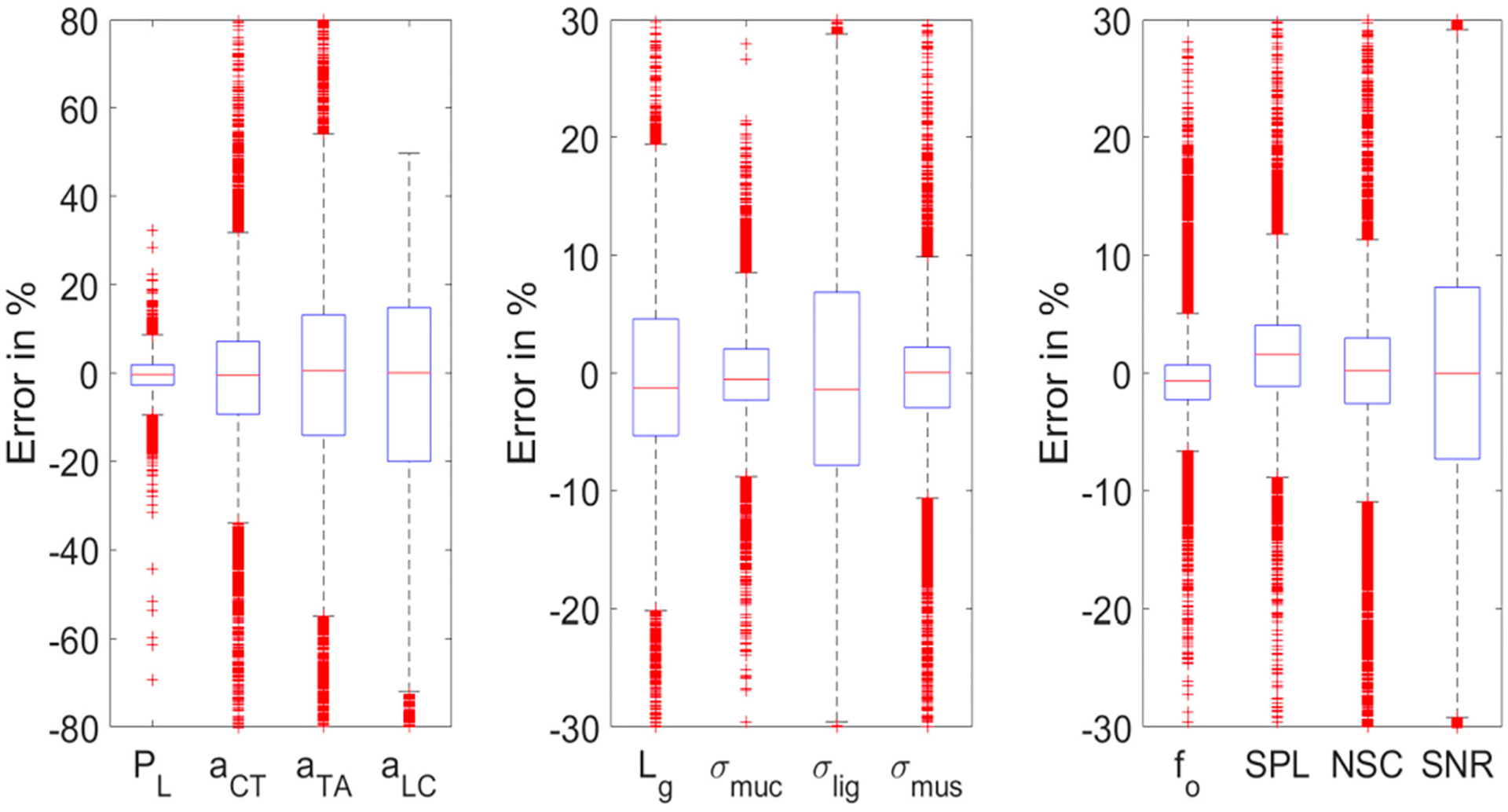
Boxplots showing error in % for the twelve features/parameters from the control system test data.

Figure 10 shows the error in % for all the twelve features/parameters from the controller test data. Like the boxplots for PtoA NN results, the *y*-axis was limited to exclude the outliers for better visibility. As observed from the individual cost values, the error in % is higher in predicting muscle targets (±80%) than somatosensory and acoustic targets (±30%), excluding outliers. For the muscle parameters, the order in error is *P*_*L*_ < *a*_*CT*_ < *a*_*TA*_ < *a*_*LC*_ according to each box’s interquartile range and the extent of whiskers. Among the somatosensory features, the error in % is moderately high for *σ*_*lig*_ (±30%), followed by L_g_ (±21%), excluding the outliers. For the other two features (*σ*_*muc*_ and *σ*_*mus*_), the error in % is low (±11%) across all the test signals, excluding the outliers. For the acoustic features, the error in % is moderately high in the prediction of SNR (±30%), and very low in the prediction of f_o_ (±7%), excluding the outliers. For the SPL and NSC, the error is relatively low (±12%).

**Figure 11.**
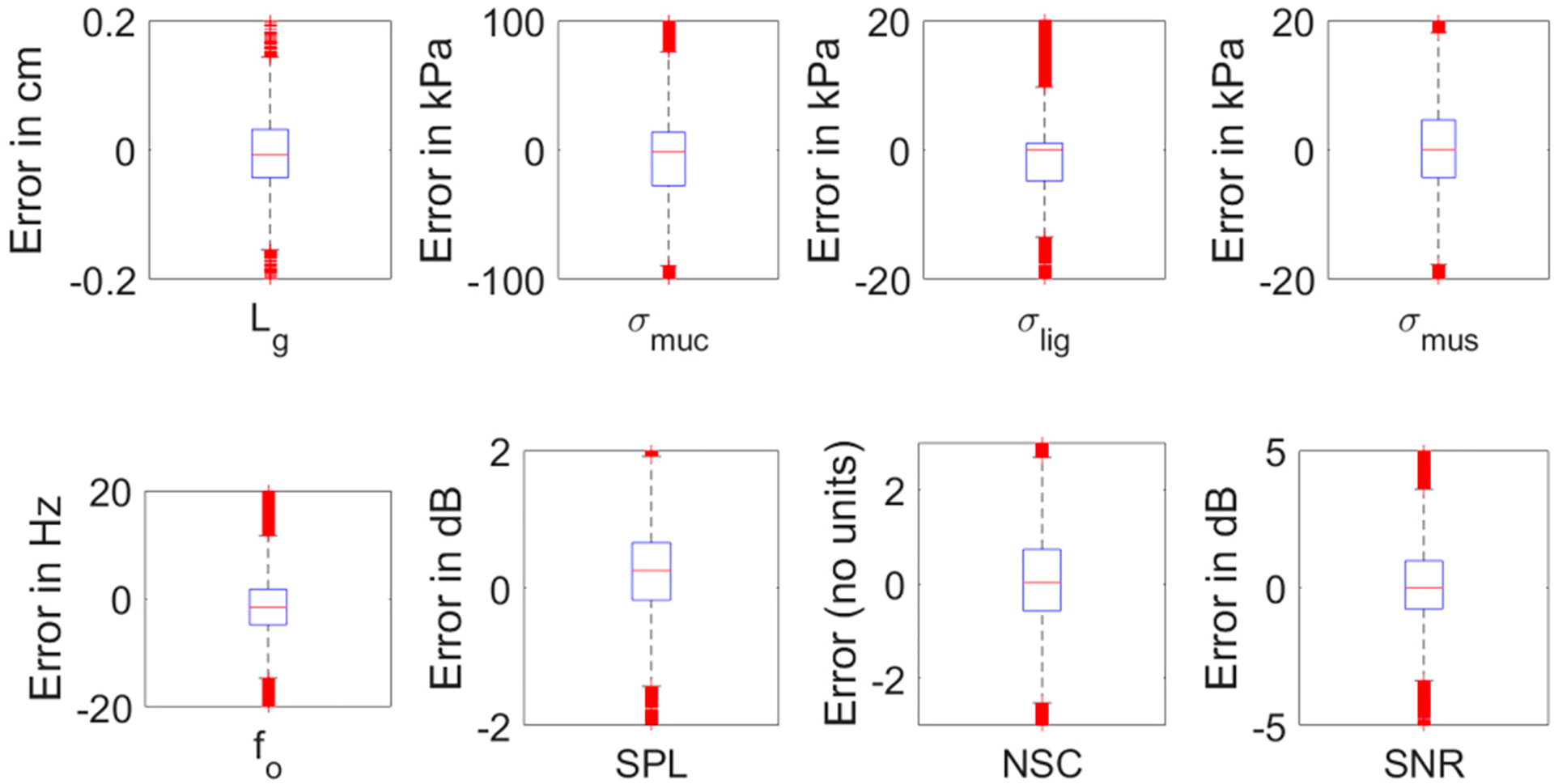
Difference between targets and predictions for (**Top row**) somatosensory features, and (**Bottom row**) acoustic features.

Figure 11 shows the difference between the targets and predictions in actual units for somatosensory and acoustic features. The control parameters were not included because they are non-dimensional, ranging between 0 and 1. The error is between ±0.2 cm for *L*_*g*_, ±100 kPa for *σ*_*muc*_, and ±20 kPa for both *σ*_*lig*_ and *σ*_*mus*_. For the acoustic features, the error is between ±15 Hz for f_o_, ±2 dB for SPL, ±3 for NSC, and ±4 dB for SNR, excluding the outliers.

**Figure 12.**
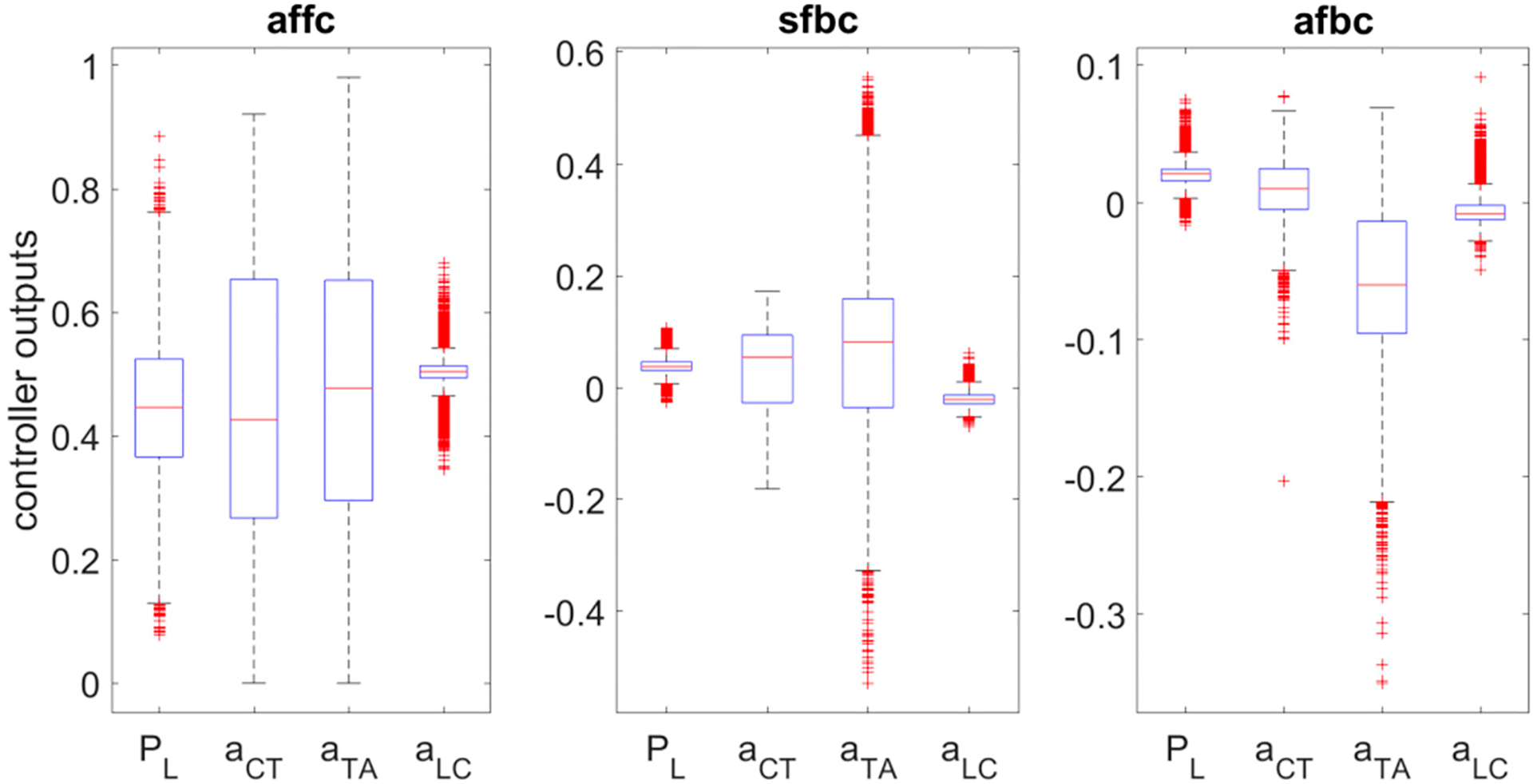
Boxplots of each controller outputs across all the test data. Here, affc—acoustic feedforward controller, sfbc—somatosensory feedback controller, and afbc—acoustic feedback controller.

Figure 12 shows the boxplot of outputs generated by each controller for the test data. It can be observed that the acoustic feedforward controller (affc) is the dominant controller after training. The range of values generated by the affc is higher than the range of values generated by the feedback controllers except for TA muscle activation. Values generated by affc ranged between 0 and 1 except for *a*_*LC*_. The values produced by affc for *a*_*LC*_ varied only in a small range around 0.5. Among the feedback controller outputs, only *a*_*TA*_ varied significantly, suggesting that more than one controller was necessary to learn the TA muscle control’s complexity.

## 4. Discussion

The current study focused on developing a neural-network-based control system for the vocal source. One acoustics feedforward controller (affc) and two feedback controllers (sfbc and afbc) were used to generate muscle activations and lung pressure to control the vocal source. A three-mass-model-based vocal system called *LeTalker* was used as the physical plant. The *LeTalker* generates somatosensory features as output, but the acoustic features should be computed from the oral pressure signal. Therefore, in the current study, a neural network (PtoA NN) was trained to compute the four acoustic features using 25 ms of the oral pressure signal. The results indicated that the PtoA NN was able to predict the four acoustic features very accurately. The error was less than ±6% for all the four acoustic features, excluding the outliers. The number of outliers ranged between 2.7% and 8.6% for the four acoustic features. A significant number of outliers in the data is expected because the lung pressure and the three muscle activations were varied randomly to generate the data. Unusual input combinations would result in several configurations that does not result in natural phonation, making the predictions extremely difficult. However, varying the input parameters randomly was necessary to explore the entire territory and make the controllers robust. The results from the controller testing suggested that, for a given target, the control system produces parameters that result in sustained phonation that is within ±15 Hz of the targeted f_o_ and ±2 dB of the targeted SPL, highlighting the stability of the control system. Previous studies found that accurate voice imitators also made errors as high as ±15 Hz during pitch imitation tasks [38], indicating excellent performance by the developed control system.

In the current study, the weights of the three controllers were updated simultaneously using a single cost function without providing any a priori knowledge about the role to each controller. Such an approach facilitated the testing of the hypothesis that the feedforward controller dominates the control after training compared to the feedback controllers. The results obtained in the current study supported the hypothesis that the range of affc outputs is higher than the range of sfbc and afbc outputs. This finding was observed in other studies as well [3,39]. Due to the inherent delays in the feedback, after training, the feedforward controller dominates, and the contribution of the feedback controllers will be minimal unless there is external disturbance [40]. This also confirms the previous observations where adults could produce speech intelligibly even after hearing loss [41]. The variation in the outputs of the feedforward controller was significantly higher than the variation in the outputs of the feedback controllers except for the TA muscle activation. This is understandable because TA muscle control is more complex compared to the other control parameters. The TA muscle changes both adduction (thyromuscularis portion of TA muscle) as well as tension (thyrovocalis portion of TA muscle) in the vocal folds.

The example waveforms indicated that the controllers could generate lung pressure and muscle activations that produced a wide variety of phonations. However, there were some differences between the targeted and predicted waveforms in terms of pitch and amplitude. It could be because of the representation of the speech signal with only four acoustic features which may not be adequate. We will address this limitation in future studies by exploring muti-parameter representation of the timbre.

Across all the test cases, the error in % was highest in reaching muscle targets (±80%). Major contributions came from *a*_*LC*_ and *a*_*TA*_. The error in % for the other two control parameters was less than ±40%. The error in % was less than ±30% for both somatosensory and acoustic features, excluding the outliers. The higher error in the prediction of control targets could be due to the nonlinearity and motor equivalence present in *LeTalker*. The *LeTalker* includes non-linear source–filter interaction phenomenon [33] described in Equation (1), which can lead to the generation of same output for multiple combinations of muscle activations. The somatosensory and acoustic features were reached with little variation in *a*_*LC*_ activation around 0.5. This could be due to the minimal dependence of the chosen acoustic and somatosensory features in the current study on *a*_*LC*_. The *a*_*LC*_ is primarily an adductor muscle, and glottal width was not considered as one of the somatosensory features in the current study. The glottal width is two-dimensional [42] and varies along the length and thickness of the vocal fold surface. It is typically measured at the level of the vocal processes (posterior end of the vocal folds). In the *LeTalker*, the steady-state glottal width was zero at the vocal processes for most of the control parameter combinations, resulting in no pattern to learn with a change in control parameters. As noticed with individual waveforms, the difference in targeted and predicted acoustic and somatosensory features could be due to the use of only four acoustic and four somatosensory features. We will explore more accurate representation of the speech waveform using acoustic features, especially for timbre, and include more somatosensory and proprioceptive features for better representation of the vocal fold posturing.

Neural networks train well on normally distributed data [43,44]. However, varying all the control parameters with normal distributions in the current study eliminated data at extreme values (i.e., values closer to 0 and closer to 1) of the muscle activations. Randomly varying all the control parameters using uniform distribution produced data even at the extremes of muscle activations. However, in the current study, the neural networks performed poorly on uniformly distributed control parameters. Therefore, the data to train and test the control system were generated by varying P_L_ and *a*_*LC*_ using normal distribution, and *a*_*TA*_ and *a*_*CT*_ using uniform distribution as a compromise. In the future, we will build the control system to overcome this limitation. Some of the somatosensory and acoustic features generated using the above-mentioned control parameters have a non-Gaussian distribution. Therefore, the acoustic features were transformed into normal distribution using a quantile transformer and the three fiber stresses were converted into logarithmic scale before training the networks. Such a transformation enhanced the performance of the neural networks.

Since the targets were constant across time in the current setup, we used feedforward artificial neural networks to represent the controllers. However, we will use recurrent neural networks in the future to accommodate time-varying targets. Since the focus of the current study was the control of vocal source, we kept the airway constant. In the future, we will include the control of the vocal tract as well for more realistic applications. Also, in the current study, we used only the acoustic and somatosensory features as targets. In the future, we will include reflex proprioceptive loops as well for more accurate representation of the control system.

We believe that this work is a steppingstone to simulate and study various motor disorders related to voice. In disorders such as Parkinson’s disease, ataxia, and flaccid paralysis, the feedforward motor program is disrupted due to damage to downstream motor control processes. It alters the established relationships between the motor commands, sensory sequences, and movement outcomes [45]. Hearing loss, muscle atrophy, or injury to the larynx can alter the feedback mechanisms associated with voice production. The control system developed in the current study can simulate such voice and sensory disorders that disrupt feedforward and feedback mechanisms and provide insights into different strategies that can be applied to restore near normal voice production. Such strategies can guide surgical interventions as well as therapy.

The utility of this work reaches beyond phonosurgery and therapy. Health scientists may be able to develop neural stimulation strategies to address motor disorders [46], while engineers may use this information in the development of prosthetics, and talking and singing robots [47]. Understanding motor learning helps voice coaches design better instruction for skill acquisition [48]. Professional vocalists will be able to improve their practice skills, along with their performances. It is therefore expected that this study will have a broad impact on voice production in humans.

## 5. Conclusions

One acoustic feedforward controller and two feedback (somatosensory and acoustic) controllers were used to generate the lung pressure and three muscle activations to produce phonation with desired acoustic targets. After training the controllers, the feedforward controller dominated the feedback controllers in generating the control parameters despite explicitly not suggesting each controller’s role. The controllers automatically learned this behavior when all the controllers were trained simultaneously using a single cost function. Due to the motor equivalence and nonlinear nature of *LeTalker*, the error in reaching muscle targets (±80%) was higher than the error in predicting the somatosensory and acoustic targets (±30%). Among the somatosensory features, the error was higher in predicting *σ*_*lig*_ than the other three features. It was observed that for most of the test cases, for a given target, the controller produces lung pressure and muscle activations that result in phonation that is within ±15 Hz of the targeted f_o_ and ±2 dB of the targeted SPL, indicating excellent performance.

## Figures and Tables

**Table 1.** Parameter values used to compute fiber stress in the three vocal fold layers.

Parameter	Mucosa	Vocal Ligament	TA Muscle
*ϵ*_1_	−0.5	−0.5	−0.5
*ϵ*_2_	−0.35	0	−0.05
*σ*_0_(kPa)	0.5	0.4	1.0
*σ*_2_(kPa)	30.0	1.39	1.5
*C*	4.4	17	6.5

**Table 2.** Control, somatosensory, and acoustic targets and their corresponding predictions for the example signals shown in Figure 9.

		Control Parameters				Somatosensory Features				Acoustic Features			
Row		P_L_	*a*_*CT*_	*a*_*TA*_	*a*_*LC*_	L_g_	*σ*_*muc*_	*σ*_*lig*_	*σ*_*mus*_	f_o_	SPL	NSC	SNR
First	Targets	0.20	0.19	0.50	0.46	1.48	12.7	8.13	12.9	129	92	10.5	−3.12
	Predictions	0.22	0.26	0.62	0.44	1.51	12.9	8.18	13.2	128	92	11.3	−4.6
Second	Targets	0.6	0.9	0.46	0.19	2.26	15.8	16.5	13.5	361	104	5.3	−0.29
	Predictions	0.63	0.95	0.45	0.41	2.24	15.75	16.4	13.5	324	101	4.25	3.1
Third	Targets	0.63	0.48	0.09	0.45	1.88	14.5	12.4	11.8	123	106	13.9	−2
	Predictions	0.65	0.44	0.11	0.47	1.84	14.4	11.8	11.9	127	106	13.3	−1.6
Fourth	Targets	0.58	0.98	0.007	0.61	2.34	16.1	17.4	12.9	269	100	3.8	13.2
	Predictions	0.57	0.96	0.0	0.43	2.38	16.2	17.8	13.0	229	95	3.5	10.9
Fifth	Targets	0.63	0.01	0.005	0.45	1.47	12.7	8.1	9.4	78	105	26.2	−7.3
	Predictions	0.68	0.04	0.17	0.49	1.42	12.4	8.0	11.8	96	105	20.7	−6.3

The control parameters were normalized. Vocal fold length has units of cm, the three fiber stress values have units of dyn/cm^2^ converted into a logarithmic scale. For the acoustic features, f_o_ has units of Hz, SPL is in dB, NSC has no units, and SNR has units of dB.

## Data Availability

The data will be provided upon request by the corresponding author. The data are not publicly available due to the complex nature of the data.
